# Electroantennographic and Behavioural Responses of European Cherry Fruit Fly, *Rhagoletis cerasi*, to the Volatile Organic Compounds from Sour Cherry, *Prunus cerasus*, Fruit

**DOI:** 10.3390/insects13020114

**Published:** 2022-01-21

**Authors:** Vincas Būda, Sandra Radžiutė, Violeta Apšegaitė, Laima Blažytė-Čereškienė, Rasa Čepulytė, Gabrielė Bumbulytė, Raimondas Mozūraitis

**Affiliations:** Laboratory of Chemical and Behavioural Ecology, Nature Research Centre, Akademijos Str. 2, LT-08412 Vilnius, Lithuania; sandra.radziute@gamtc.lt (S.R.); violeta.apsegaite@gamtc.lt (V.A.); laima.blazyte@gamtc.lt (L.B.-Č.); rasa.cepulyte@gamtc.lt (R.Č.); gabriele.bumbulyte@gamtc.lt (G.B.); raimondas.mozuraitis@gamtc.lt (R.M.)

**Keywords:** volatiles, kairomone attractants, EAG, GC–MS, olfactometry

## Abstract

**Simple Summary:**

The larvae of European cherry fruit flies are developing in sweet and sour cherry fruit. Non-insecticidal methods to control this pest are needed since most of the conventional insecticides used have been banned in Europe. Mass trapping is one of the environmentally friendly methods, however, it requires highly effective pest attractants. Three volatile compounds were identified as attractive to females of this species.

**Abstract:**

European cherry fruit fly, *Rhagoletis cerasi* (Diptera: Tephritidae), is the most important pest of sweet and sour cherry fruit. This fly is difficult to control by insecticide application since most of the conventional insecticides used have been banned in Europe. Traps are used for both the pest’s mass trapping and the detection of the beginning of the flight period. Data on flies’ reactions to host-plant volatile organic compounds (VOCs) can be used to search for new attractants. VOCs were collected from the headspace of sour cherry, *P. cerasus*, fruit. Gas chromatography–mass spectrometry (GC–MS) resulted in the identification of 51 compounds. Terpenes and esters predominated in two aspects: in the highest diversity of the compounds, and the amount of the total VOC emissions (62.3%). Among the single VOCs, ethyl octanoate prevails, followed by (*E*)-4,8-dimethyl-1,3,7-nonatriene. GC–electroantennographic detection (GC–EAD) revealed 14 EAG-active compounds and those were identified. In Y-tube olfactometer tests, EAG-active compounds ((*E*)-*β*-ocimene, linalool, and (*Z*)-3-hexenyl 3-methylbutanoate) attracted *R. cerasi* females in a similar way to the odour of sour cherry fruit.

## 1. Introduction

European cherry fruit fly *Rhagoletis cerasi* L. (Diptera: Tephritidae), is an economically important frugivorous pest of sweet, *Prunus avium*, L., and sour, *Prunus cerasus* L., (Rosales: Rosaceae) cherries, common all over Europe, East Asia, and since 2016–2017 the pest has been recorded in North America [1,2]. Females lay their eggs on the cherry fruit and larvae develop inside them. *R. cerasi* is considered a key pest that requires insecticide sprays several times per season in commercial cherry orchards (e.g., [3]), when adult fruit flies start flying. In many countries insecticides are increasingly restricted and most of those used previously to control cherry fruit flies have been banned in Europe (e.g., [4]). Pest control of sweet and sour cherries is especially challenging since most of the fruit are delivered to the market immediately after harvest, and this significantly restricts management options. Meanwhile, even a small percentage of cherries infested with larvae is unacceptable to the consumer. Because sweet and sour cherries are important not only as a food product but also from a medical point of view [5], production without the insecticide applications (under organic farming conditions) is particularly valuable. Environmentally-friendly pest control methods have been proposed such as application of nematodes to the soil (e.g., [6]), spraying cherry trees and fruit with entomopathogenic fungus *Beauveria bassiana* biopreparations [7,8] or applying a dense mesh on the undergrowth soil [3,9] thus prevent emerging adults from getting to the host plants and fruit. Another way of control is using traps with attractants for adult mass trapping (e.g., [10,11,12]). Yellow ball-shaped traps supplied with ammonium salts as attractants inside are the most effective. However, mass trapping of *R. cerasi* so far is too expensive and was estimated from $2500 to $3500/ha [12]. To reduce the cost, more effective trapping tools are needed that would decrease the amount of attractant used. Attractants effective for females would be of special interest, as this would prevent fruit damage by their offspring. We hypothesise that host-plant volatile organic compounds (VOCs) can be identified that increase the attraction of *R. cerasi* females to traps.

## 2. Materials and Methods

### 2.1. Insects

Overwintered puparia of fruit fly *R. cerasi* were collected from the soil under sweet and sour cherry trees during April-May of 2019–2021 in Vilnius (GPS coordinates: 54°45′24.0″ N 25°03′02.4″ E) and Kaunas (GPS coordinates: 54°54′13.8″ N 23°48′07.9″ E), Lithuania. Each puparium was placed into a separate 14 mL glass vial containing wet 3 cm^2^ filter paper inside and corked by a foam stopper. The vials were kept at 20–24 °C, 16 L: 8 D (light:dark) photoperiod and 65–75% relative humidity in a climate chamber “Fitotron” (Weiss Gallenkamp, UK). To maintain high humidity inside a vial, 2–3 drops of water were added to the filter paper twice a week. After approximately 10–14 days on average, emerged adults were transferred to a walk-in climate room under 18–20 °C, natural daylight photoperiod, 50–60% relative humidity and were kept in the same vials. For feeding, 10% sugar solution in water was provided. The flies were sexed visually based on the presence or absence of an ovipositor and were kept under the same conditions as indicated above.

### 2.2. Cherry Fruit

*Prunus cerasus* fruit of the local variety were collected aseptically in the beginning of July 2018 in a private orchard located in Rastinėnai, Vilnius region, Lithuania (GPS coordinates: 54°45′24.0″ N, 25°03′02.4″ E). The fruit were immediately transferred to the laboratory for VOC collection and bioassay.

### 2.3. Chemicals

The following compounds were purchased from Sigma–Aldrich (St. Louis, MO, USA): hexanal (98% chemical purity), (*E*)-2-hexenal (98% chemical purity), octanal (99% chemical purity), nonanal (95% chemical purity), decanal (≥98% chemical purity), (*E*)-2-nonenal (97% chemical purity), benzaldehyde (≥99% chemical purity), 6-methyl-5-hepten-2-one (99% chemical purity), 1-hexanol (≥99% chemical purity), (*Z*)-3-hexen-1-ol (98% chemical purity), 1-octen-3-ol (98% chemical purity), propyl 3-methylbutanoate (≥98% chemical purity), 3-methylbutyl propionate (≥98% chemical purity), 3-methylbutyl 3-methylbutanoate (≥98% chemical purity), hexyl acetate (99% chemical purity), ethyl octanoate (≥99% chemical purity), (*Z*)-3-hexenyl acetate, (*Z*)-3-hexenyl 3-methylbutanoate (≥97% chemical purity), *α*-pinene (98% chemical purity), *α*-phellandrene (≥95% chemical purity), limonene (≥98% chemical purity), (*E*)-*β*-ocimene (≥90% chemical purity), *p*-cymene (99% chemical purity), linalool (97% chemical purity), *α*-terpinyl acetate, 2-phenylethyl acetate (99% chemical purity), (*E*)-geranyl acetone (96% chemical purity), hexane (≥99% chemical purity). Ethyl hexanoate (99% chemical purity), 3-methylbutyl acetate (98% chemical purity) were purchased from Alfa Aesar (Ward Hill, MA, USA), *δ*-3-cerene from Carl Roth (Karlsruhe, Germany), α-farnesene from Bedoukian Research Inc. (Danbury, CT, USA).

### 2.4. Collection of Fruit Volatiles

Headspace volatiles were collected from 240 g *P. cerasus* fruit in a 350 mL glass flask fitted with an inlet for purified air at 0.5 L/min flow and an adsorbent trap containing 150 mg of Porapak Q 50–80 mesh (Supelco, Darmstadt, Germany), in a 60 mm long × 5 mm diameter glass tube. For a collection of control samples, a charcoal-filtered air stream was pulled through an empty glass flask. VOCs were collected for 24 h, eluted with 3 × 250 µL of *n*-hexane, and concentrated to 200 µL under a gentle nitrogen stream. Three collected samples were combined and stored at −18 °C until analysed.

### 2.5. Gas Chromatography–Electroantennogram Detection

A gas chromatography–electroantennogram detection (GC–EAD) system consisting of Clarus 500 gas chromatograph (PerkinElmer, Waltham, MA, USA) equipped with a flame ionization detector (FID) and DB-Wax capillary column (30 m × 0.25 mm × 0.25 µm; Agilent Technologies, Santa Clara, CA, USA) coupled with an electroantennogram detection device (Syntech IDAC 4, Hilversum, The Netherlands) was used. The temperature was set at 240 °C both in the injector and the detector. The oven temperature was maintained isothermally at 40 °C for 2 min afterwards, it was raised to 200 °C at a rate of 5 °C/min, then increased to 240 °C at a rate of 10 °C/min and maintained isothermally for 2 min. Hydrogen at a flow rate of 1.5 mL/min was used as a carrier gas. At the end of the column, an effluent was divided by a splitter into two parts allowing simultaneous FID and EAD detection of the separated VOCs. A nitrogen make-up gas at a flow rate of 5 mL/min was used to enhance FID performance. One part of the split effluent was directed into a purified and humidified air stream flowing at 0.5 m/s through a glass tube over fruit fly antennal preparation.

For GC–EAD analyses just females, not chilled, or otherwise anaesthetised, were used. The fly was fixed by putting into a plastic tip (yellow, Eppendorf, Germany) and immobilised by cotton plug. The end of the tip was cut to allow the insect’s head to slip through it, and the head was severed at the base with Vannas spring scissors (Fine Science Tools, Heidelberg, Germany). A glass capillary indifferent electrode filled with Ringer solution (Fresenius Kabi, Warsaw, Poland) and grounded via a silver wire was inserted into the base of the severed head of the fly. The recording electrode, connected to a high-impedance DC amplifier with automatic baseline drift compensation, was a similar capillary, brought into contact with the distal end of the fly antenna. The antennal and the FID signals were recorded simultaneously, stored, and analysed using software GcEad V. 4.4 (Synthech, NL 1998). Before injection of a sample, the condition of the antenna was tested by stimulation with 3-methylbutan-1-ol (0.1 mg/mL). Each EAD recording was replicated 12 times, and each antenna was tested only once.

### 2.6. Gas Chromatography–Mass Spectrometry

For gas chromatography–mass spectrometry (GC–MS) analysis Shimadzu gas chromatograph GC-2010 coupled with Shimadzu MS-QP 2010 Plus mass selective detector (Shimadzu, Japan) was used. The GC was equipped with a DB-Wax column (30 m × 0.25 mm × 0.25 µm, Agilent Technologies, Santa Clara, CA, USA) and operated under the same conditions as indicated in the methods for GC–EAD detection, except that carrier gas was helium, and the flow rate 1.5 mL/min. Electron ionization spectra of the compounds were acquired at an electron energy of 70 eV, and the interface and ion source temperatures were held isothermal at 240 °C. Each VOC emitted by sour cherry fruit was identified by comparison either of its mass spectral data and retention indices with the corresponding data available from NIST version 2.0 mass spectral search programme (National Institute of Standards and Technology, USA) or by comparison with those of synthetic standards (indicated in the Table 1) using the software GCMS solution version 2.71.

### 2.7. Behavioural Test

Y-olfactometer was used to assess the behavioural responses of *R. cerasi* females to the headspace VOCs of cherry fruit and to evaluate the attractiveness of five EAG-active VOCs: (*E*)-*β*-ocimene, 3-methylbutyl 3-methylbutanoate, (*Z*)-3-hexenyl 3-methylbutanoate, linalool, and (*E*)-geranyl acetone. A Y-type olfactometer (the main Y-tube length 25 cm; two branches of 17 cm length; the angle between the branches 110°; inner diameter of each branch 5 cm) was placed in a fume cupboard. Four 18 W cylindrical white lamps (LT 18W T8/840, Colourlux plus, NARVA, Brand-Erbisdorf, Germany) covered with plastic, white, matt shield (65 cm long, 42 cm wide) were installed in front of the olfactometer at a distance of 23 cm. A flow of clean (filtered) air at a rate of 0.6 L/min was split and passed through two glass vessels containing either the odour source or control stimuli and entered each of the branches of the Y-tube. Air supply system CADS-4CPP (Sigma Scientific LLC, Micanopy, FL, USA) was used for air filtration and flow rate control.

For assessment of the fruit fly females’ response to the cherry fruit headspace VOCs, four light red cherry fruit on a twig were placed in 300 mL glass flask connected to one branch of the Y-tube. For the control a similar twig with no fruit was placed into another glass flask joined to the other branch of the olfactometer. Due to the configuration of the olfactometer and position of the flasks direct visual fruit fly stimulation by fruit image was excluded.

To assess the attractiveness of the synthetic VOC, 10 µL of the test compound at a concentration of 0.1 mg/mL was spread on a filter paper (5 × 40 mm). The concentration was chosen based on the previous *R. cerasi* olfaction study (Mozūraitis et al., in press). For control, 10 µL of solvent (hexane) was used. Thirty seconds after loading and solvent evaporation, both the filter paper containing the test compound and the filter paper containing the solvent (control), were placed into separate glass tubes (length 7 cm, diameter 0.7 cm) for the odour delivery into the corresponding branch of the Y-olfactometer.

Mated *R. cerasi* females aged 5–14 days were tested. To ensure that mated females were used in the behavioral assays, a day before the test two males were put into an individual vial with a female and left for 24 h. Only females that mated were selected for the tests. Females were fed a 2% sugar solution prior to the test. Each fly was placed individually in the olfactometer and observed for 10 min. The VOC choice was recorded if the fly reached the glass container with the odour source. In case a fly returned from the Y-tube branch with VOC not reaching the glass container with the odour source and moved to an alternative branch of the olfactometer and reached the vessel with the control stimulus there, the selection was recorded as the final (VOC negative) one. If within 10 min any branch of the Y-tube was not chosen, the fly was considered inactive and was excluded from the test. The position of the olfactometer’s branches was swapped regularly after testing 5 females. The test was performed during a day period (between 10 am and 5 pm) at a temperature of 23 ± 2 °C and a humidity of 60%. Each *R. cerasi* female was used only once for testing.

After testing a single chemical, the olfactometer was disassembled, glass parts were washed with hexane, soaked in distilled water, and heated in a thermostat at 200 °C. The silicone parts were processed the same way except heating and were dried at room temperature.

Before the start of the choice tests, a control test was performed. Air stream carrying pure air in both branches of the olfactometer was used. The control test checked whether the insects chose the right and left branches of the olfactometer equally often, i.e., whether light and other environmental conditions were properly balanced. The tests were started following correct balance only (when fruit flies selected both branches of the olfactometer at the same frequency).

Statistical analysis of the results was performed using the χ2 test of the software STATISTICA 7.0 (StatSoft, Tulsa, OK, USA).

## 3. Results

### 3.1. Cherry Fruit VOCs

Fifty-five compounds were isolated in sour cherry, *P. cerasus*, fruit headspace. Of these, 51 VOCs have been identified (Table 1). Identified compounds were attributed to seven groups: alkanes (7 VOCs), aldehydes (9 VOCs), esters (12 VOCs), alcohols (6 VOCs), ketones (2 VOCs), and terpenes (15 VOCs). Terpenes and esters predominated among the volatiles. These two groups of compounds predominated in two aspects: the highest diversity of the compounds and the highest abundance in VOC emissions. In total, those accounted for 62.2% of VOC emissions (terpenes 33.4 and esters 28.8%, respectively). Alkanes composed 13.2%, aldehydes 13.1%, alcohols 5.7%, and ketones each 1.7% of the total VOC content. Among the single components, ester ethyl octanoate prevailed in abundance most prominently (Table 1), followed by homoterpene (*E*)-4,8-dimethyl-1,3,7-nonatriene (approximately 2.5 times less abundant).

### 3.2. Olfactory Active VOCs

**Figure 1:**
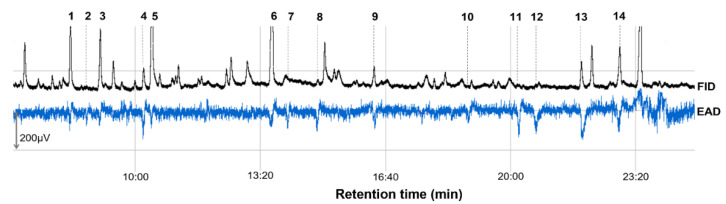
Example of GC–EAD responses of fruit fly female, *Rhagoletis cerasi*, to the volatile compounds from the headspace of sour cherry, *Prunus cerasus*, fruit/FID, flame ionisation detector, EAD, electroantennographic detector, DB-Wax capillary column (30 m × 0.25 mm × 0.25 µm; Agilent Technologies, Santa Clara, CA, USA), FID peaks are numbered according to Table 2.

GC–EAD recordings revealed the presence of 14 active peaks in antennal responses of European cherry fruit fly females to volatile compounds released by sour cherry fruit (Figure 1). This indicated that female antenna is sensitive to 14 VOCs at least. Results of EAG-active compound identification by GC–MS alongside with their retention time and other characteristics are presented in Table 2.

It should be noted that the variation in amounts among the EAG-active compounds sampled from the headspace of cherry fruit was large. They fit into a few groups according to abundance. The most abundant compounds, where each VOC accounts for more than 10% of the total mixture of EAG-active compounds, include: (*E*)-2-hexenal, (*E*)-*β*-ocimene, and (*E*)-4,8-dimethyl-1,3,7-nonatriene. Only a single compound fits the range of abundance from 5 to 10 %, namely (*E*)-geranyl acetone. The group where each compound composes up to 5% of the mixture includes four VOCs: 3-methylbutyl 3-methylbutanoate, linalool, 2-phenylethyl acetate and ethyl octanoate. Although the relative content of the latter compound (as indicated in Table 1) is much higher, we noted that this result was obtained due to contamination with this compound during the sample preparation stage. Therefore, we assign the compound to this group of abundance approximately. The least abundant compounds (<1% each) are the following six VOCs: ethyl hexanoate, 1-octen-3-ol, (*Z*)-3-hexenyl 3-methylbutanoate, unknown compound, *α*-muurolene, and *α*-farnesene.

### 3.3. Behavioural Response to Fruit

**Figure 2:**
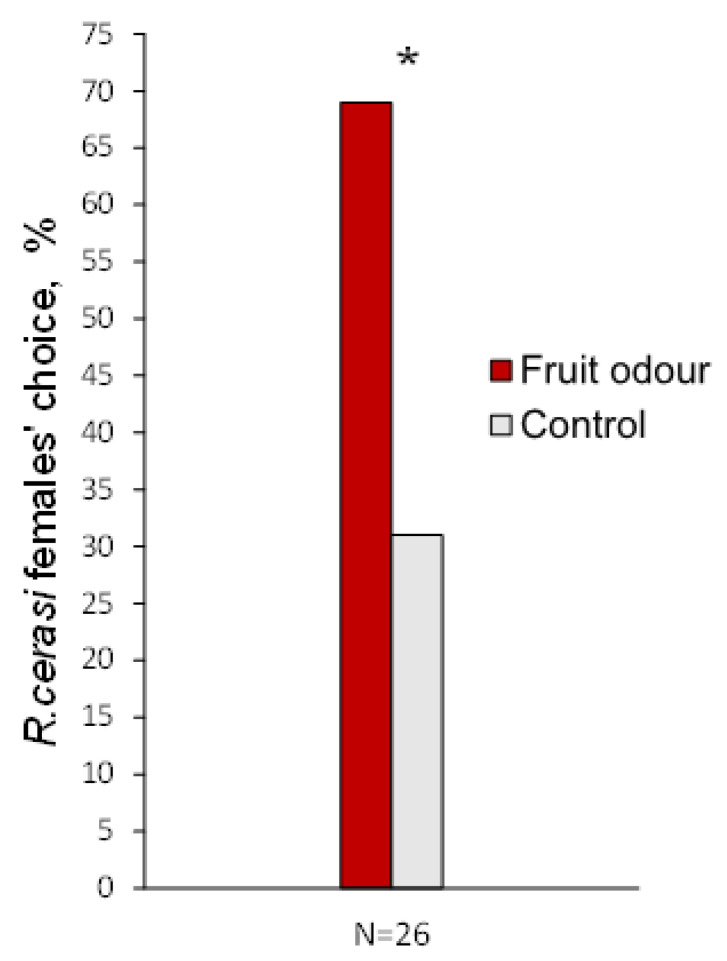
*Rhagoletis cerasi* females’ choice of cherry fruit odour versus control in Y-olfactometer. Asterisk marks a statistically significant difference (* *p* < 0.05).

To evaluate the suitability of behavioural assay, before starting the search for behaviourally active cherry fruit VOCs, we checked *R. cerasi* female reaction to the fruit in the olfactometer. The airflow carrying the smell of cherry fruit was chosen by 69% of fruit fly females tested, and 31% of females entered the alternative airflow without the smell (Figure 2). This difference was significant (*p* < 0.05), and this led to the conclusion that both the environmental conditions and the device used for testing were appropriate to look for behavioural effects of a single VOCs.

### 3.4. Behavioural Reaction to Single VOCs

**Figure 3:**
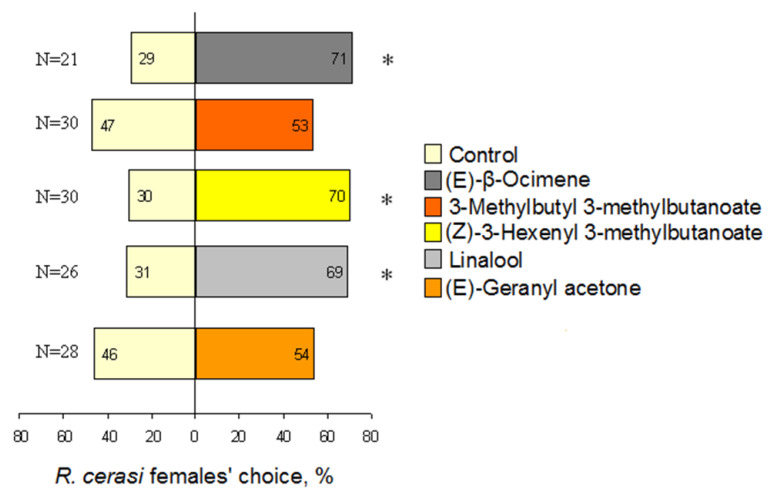
Behavioural response of *Rhagoletis cerasi* fruit fly females to odorants in Y-olfactometer. Significant difference (* *p* < 0.05) is marked with an asterisk.

Based on preliminary observations and literature analysis, the following five EAG-active compounds were selected for behavioural testing: (*E*)-*β*-ocimene, 3-methylbutyl 3-methylbutanoate, (*Z*)-3-hexenyl 3-methylbutanoate, linalool, and (*E*)-geranyl acetone. When testing single compounds, three of them were found attractive for *R. cerasi* females: two terpenes, (*E*)-*β*-ocimene and linalool, as well as ester (*Z*)-3-hexenyl 3-methylbutanoate (Figure 3). The attractivity of these compounds in the olfactometer differed from that of the control significantly (*p* < 0.05), thus each of these three host-plant VOCs is attributed to kairomone attractants, following behaviourally active compound classification accepted in chemical ecology. Behaviour of *R. cerasi* females turned out to be indifferent in presence of the odours of the two compounds: ester 3-methylbutyl 3-methylbutanoate and terpene (*E*)-geranyl acetone, those were neither attractive nor repellent (Figure 3).

## 4. Discussion

The compounds emitted by the sour cherry fruit so far have been studied very fragmentedly, therefore the chemical composition has been poorly investigated. The largest number of volatile compounds, i.e., 12, were identified by Levaj et al. [13]. They include alcohols, terpenes, and aldehydes (three compounds in each group); other VOCs found belong to esters (2) and ketones (1). Of these 12 compounds, five were detected in the present study as well. These were: hexan-1-ol, benzaldehyde, hexanal, (*E*)-2-hexenal, and linalool. According to Levaj et al. [13], sour cherry fruit emissions contain the highest amount both of benzaldehyde and linalool, which is different from the results we have obtained: benzaldehyde was among the minor components, while linalool was below the top 10 VOCs in terms of abundance (Table 1). Low similarity with our results could be due to several reasons: different varieties of sour cherry analyzed (it is well-known that VOC composition varies depending on the cherry variety, e.g., [13,14], different techniques used for the collection of VOCs, and the remarkably different temperature regimes used for the collection of compounds. We sampled VOCs at room temperature (approximately corresponding to that in nature), while [13] collected VOCs from homogenised samples of fruit and jam heated up to 50 °C for half an hour. Under such conditions, many volatile compounds have been lost and the relative quantities of the identified ones do not reflect their proportion present under natural conditions. So, as far as we know, the 39 compounds emitted by intact sour cherry fruit were reported for the first time in the present paper.

It is interesting to compare the VOC composition of sour cherry, *P. cerasus*, fruit and that of closely related sweet cherry, *P. avium*. Sweet cherry fruit emissions have been studied in more detail, but in most cases, the fruit have been homogenised [15,16], frozen (e.g., [16,17]), stored before analysis mimicking commercial practice [18] or otherwise processed. Only one work provides data on *P. avium* VOCs from intact fruit [19], so for comparison with our results, we chose exactly this data.

In the present study, 51 VOCs were identified from fruit of sour cherry, *P. cerasus*, while Mattheis et al. [19] reported 28 volatiles released by fruit of sweet cherry, *P. avium*. Only 11 compounds were common: hexanal, octanal, nonanal, decanal, benzaldehyde, hexan-1-ol, 2-ethylhexan-1-ol, 3-methylbutyl acetate, ethyl hexanoate, hexyl acetate and ethyl octanoate. Predominant compound groups differed as well: esters and terpenes accounted for 62.2% of VOCs in *P. cerasus,* while alcohols and aldehydes composed approximately 88.2% in *P. avium* VOCs. The most abundant compounds were: ester, ethyl octanoate followed by homoterpene, (*E*)-4,8-dimethyl-1,3,7-nonatriene, in *P. cerasus*, while two alcohols, 2-propanol, and ethanol, in *P. avium.*

Such pronounced differences are likely to be not only due to objective interspecific characteristics of VOC emissions but in particular, due to different methods applied both during fruit preparation for analysis and volatile collection.

Data on the effects of sour cherry released chemicals regarding fruit fly *R. cerasi* adults so far have not been published. Even for the other species within the genus *Rhagoletis*, this data is very scarce, except for the only species, apple maggot fly, *R. pomonella* [20,21].

It is known that following adult hatching, fruit fly adults from the family Tephritidae are searching for food, necessary for the development of reproductive glands and sexual maturation. Tephritids are attracted by volatile ammonia-based compounds that are associated with protein decomposition and amino nitrogen [22]. One of such compounds, tested on European cherry fruit flies, was ammonium acetate [23], either as a single compound or mixed with other ammonium salts and additive 1,4-diaminobutane (putrescine) [24], a nitrogen-containing product of breakdown of amino acids. These compounds affect fruit fly behaviour as placed inside traps increase *R. cerasi* catches compared to control, but their effectiveness still remains low [25]. It can be assumed that when adults reach sexual maturity in a short period of time after emergence, their motivation changes, and instead of feeding attractants, sex attractants become more important.

Analysis of chemical compounds in volatiles and extracts of males *R. cerasi*, revealed the presence of 75 compounds [26]. Some of those could function (or mimic) sex pheromone, and two baits each loaded with three component mixtures: of 2-hexanone, 3-heptanone and nonanal as well as of *β*-phellandrene, geranyl acetate, and (+)-limonene attracted up to three times more fruit flies compared to control in the field trapping tests [27]. Since all these compounds have been found in *R. cerasi* males’ extracts/volatiles [26], it was speculated that at least some of them could influence fruit fly behaviour as related to searching for a mating partner [27].

After the fruit fly females are mated, their motivation should change from sexual to reproductive (egg-laying). Thus, at this stage, fruit should become attractive, i.e., their emitted VOCs alongside with visual stimuli [4]. Because, as we mentioned above, VOCs of sour cherry fruit attractive to *R. cerasi* females were not known, our established three behaviourally active compounds, namely (*E*)-*β*-ocimene, linalool and (*Z*)-3-hexenyl 3-methylbutanoate are new kairomone attractants. Below we discuss all the revealed olfactory-active compounds, their influence on the behaviour of fruit flies related to European fruit fly *R. cerasi*, those within the genus *Rhagoletis*. In the absence of data at the genus level, results from the Tephritidae family are provided. In the absence of such data at the family level, results from the order Diptera or other taxa are provided. In all cases, we used data provided in the Pherobase database [20], if another source, it is indicated in brackets as quoted literature.

(*E*)-2-hexenal. The compound is well known as a green leaf component. It is known to affect the behaviour of six Dipteran species from three families: it is attractive as a single compound to flies *Milichiella arcuata* and *Paramya nitens* and as an admixture for another species (all from family *Melechiidae*) as well as for three species from *Chloripidae* family and one species from the *Psillidae* family.

Ethyl hexanoate. The compound is reported as a pheromone component for *Anastrepha striata*, *A. ludens* and *A. fraterculus* fruit flies and as an attractant for two species from the same family (all belong to the family *Tephritidae*).

(*E*)-*β*-Ocimene. The volatile functions as the pheromone component of *Anastrepha fraterculus* (*Tephritidae*) and attractant for flies of four dipteran species from families *Chloropidae* and *Lauxaniidae* as an admixture with some other compounds.

3-Methylbutyl 3-methylbutanoate. We are not aware of any data on the behavioural activity of this compound in dipterans.

(*E*)-4,8-Dimethyl-1,3,7-nonatriene. The compound attracts maggot fly, *R. pomonella,* and functions as a kairomone component of the host plant [28], as well as a component in an attractive mixture to *R. zephyria* [29].

Ethyl octanoate. The ester is behaviourally active towards the following Tephritids: as a pheromone component to *Anastrepha striata*, as an attractant (in a mixture) to *A. obliqua* and *A. ludens*.

1-Octen-3-ol. Kairomone component of tephritid species *R. pomonella*, *R. zephyra* and *Bactrocera dorsalis*, an oviposition stimulant in the latter species.

(*Z*)-3-Hexenyl 3-methylbutanoate. No behavioural effect for dipterans was recorded, but the VOC is known as attractant for two species of Hymenopterans (from *Braconidae* and *Melittidae* families).

Linalool. The compound causes behavioural reactions in fruit flies of the three Tephritidae species at least: as a pheromone component of *Anastrepha striata*, as a component of attractive mixture to *R. zephyria*, and as a deterrent to the Mediterranean fruit fly *Ceratitis capitata* [30].

α-Muurolene. Floral VOC of many plant species, neither as an attractant nor repellent for Tephritid fruit flies was reported.

α-Farnesene. Known as pheromone component of five species from Tephritidae family: *Anastrepha fraterculus*, *A. ludens*, *A. obliqua* and *A. suspensa*, *Ceratitis capitata* as well as oviposition stimulant for *Bacrtocera dorsalis*.

2-Phenylethyl acetate. The behavioural role in dipterans was not recorded.

(*E*)-Geranyl acetone. Widespread attractant component in coleopterans. Among dipterans is known as a component of an attractive mixture in a single species only (mosquito *Anopheles gambiae*).

Thus, of the 14 EAG-active for *R. cerasi* fruit flies’ compounds, reported in the present paper, seven are known to influence behaviour of other fruit flies from the genus *Rhagoletis*. Among them, five chemical compounds function as pheromones of Tephritidae species occurring outside Europe. The latter circumstance raises an interesting question about the origin of these semiochemicals. Since they are secreted by both the plants (e.g., the present study) and the insects [20], the question arises as to whether the insects synthesise the VOCs by themselves or use those accumulated from the plants while feeding at the larval stage. Such overlap of VOCs could be the product of common metabolic processes as well.

Based on the material presented, one may conclude that *R. cerasi* and related species, discussed above, share common systems for certain odorants’ perception. This provides additional information on evolutionary relationships within the family Tephritidae.

It should be noted that among the VOCs collected from sour cherry fruit (Table 1), including those to which EAG reactions of *R. cerasi* fruit flies have been recorded (Table 2), there are compounds known as produced by fruit-related yeasts. For example, among the latter compounds, there are VOCs released by the yeast *Pichia kudriavzevii*: 3-metylbutyl acetate, ethyl hexanoate, ethyl octanoate, 2-ethylhexan-1-ol, 3-methylbutyl propionate, and 2-phenylethyl acetate [31].

The issue on the relation of VOCs of plant and/or microorganism origin and those, which function as pheromones in Tephritidae fruit flies (compounds released by insects) should be thoroughly investigated in the future.

## 5. Conclusions

The application of insecticides is undesirable, and conventional insecticides used to control European cherry fruit flies, *Rhagoletis cerasi*, are even banned in Europe. Among the alternative environmentally friendly cherry fruit fly control methods mass trapping has been proposed. To implement this technology the most effective attractant is required. This study investigated cherry fruit fly reactions to the volatile organic compounds (VOCs) released by sour cherry, *Prunus cerasus*, fruit. In VOCs collected from cherry fruit headspace under natural conditions, 54 compounds were isolated, and 51 were identified. Among these, 14 were olfactory active to females of *R. cerasi*. Three of them were attractive under laboratory conditions: (*E*)-*β*-ocimene, linalool, and (*Z*)-3-hexenyl 3-methylbutanoate. The compounds are the first kairomone attractants for *R. cerasi* fruit flies and can be used either as single compounds or as a mixture/admixture with other attractants to increase the efficiency of trap attractiveness.

## Figures and Tables

**Table 1:** Volatile organic compounds from the headspace of sour cherry, *Prunus cerasus*, fruit identified by GC–MS.

No	Compound	RI ^1^	CAS No ^2^	Group ^3^	ID ^10^	Abundance, %
1	n-Decane	1000	112-40-3	A ^4^	RC ^11^	0.71
2	*α*-Pinene	1016	80-56-8	T ^5^	RC	1.15
3	Hexanal	1075	66-25-1	AL ^6^	RC	0.65
4	n-Undecane	1100	1120-21-4	A	RC	0.56
5	3-Methylbutyl acetate	1117	123-92-2	E ^7^	RC	0.43
6	δ-3-Carene	1141	13466-78-9	T	RC	0.79
7	Propyl 3-methylbutanoate	1151	557-00-6	E	RC	0.36
8	α-Phellandrene	1158	99-83-2	T	RC	3.08
9	3-Methylbutyl propionate	1184	105-69-0	E	RC	0.07
10	Limonene	1189	5989-27-5	T	RC	7.60
11	*β*-Phellandrene	1196	555-10-2	T	L ^12^, RI	0.36
12	n-Dodecane	1200	112-40-3	A	RC	0.87
13	(*E*)-2-Hexenal	1207	6728-28-3	AL	RC	3.51
14	Ethyl hexanoate	1224	123-66-0	E	RC	0.16
15	(*E*)-β-Ocimene	1247	3779-61-1	T	RC	4.66
16	*p*-Cymene	1261	99-87-6	T	RC	1.88
17	Hexyl acetate	1268	142-92-7	E	RC	0.61
18	Octanal	1281	124-13-0	AL	RC	0.56
19	3-Methylbutyl 3-methylbutanoate	1289	659-70-1	E	RC	1.85
20	(*E*)-4,8-Dimethyl-1,3,7-nonatriene	1299	19945-61-0	T	L, RI	9.90
21	(*Z*)-3-Hexenyl acetate	1308	3681-71-8	E	RC	2.35
22	(*Z*)-2-Hexenyl acetate	1327	56922-75-9	E	L, RI	0.98
23	6-Methyl-5-hepten-2-one	1328	110-93-0	K	RC	1.10
24	1-Hexanol	1349	111-27-3	OH ^8^	RC	0.53
25	Unknown	1368				0.93
26	(*Z*)-3-Hexen-1-ol	1378	928-96-1	OH	RC	0.82
27	Nonanal	1385	124-19-6	AL	RC	2.83
28	n-Tetradecane	1400	629-59-4	A	RC	3.47
29	Ethyl octanoate	1429	106-32-1	E	RC	17.82
30	Unknown	1441				2.12
31	1-Octen-3-ol	1446	3391-86-4	OH	RC	0.18
32	(*Z*)-3-Hexenyl 3-methylbutanoate	1480	35154-45-1	E	RC	0.53
33	2-Ethylhexan-1-ol	1484	104-76-7	OH	RC	3.03
34	Decanal	1490	112-31-2	AL	RC	2.44
35	n-Pentadecane	1500	629-62-9	A	RC	3.08
36	Benzaldehyde	1502	100-52-7	AL	RC	0.87
37	(*E*)-2-Nonenal	1517	78-70-6	AL	RC	0.70
38	Linalool	1541	78-70-6	T	RC	1.89
39	n-Hexadecane	1600	544-76-3	A	RC	2.61
40	6-Methylheptan-1-ol	1609	1653-40-3	OH	L, RI	0.74
41	Unknown	1623				1.02
42	Acetophenone	1630	98-86-2	K ^9^	L, RI	0.57
43	1-Nonan-1-ol	1661	143-08-08	OH	L, RI	0.39
44	Unknown	1664				0.05
45	*α*-Terpinyl acetate	1683	80-26-2	T	RC	0.24
46	3-Ethylbenzaldehyde	1688	34246-54-3	AL	L, RI	1.10
47	Germacrene D	1693	23986-74-5	T	L, RI	0.39
48	n-Heptadecane	1700	629-78-7	A	RC	1.94
49	*α*-Muurolene	1711	17627-24-6	T	L, RI	0.15
50	4-Ethylbenzaldehyde	1717	4748-78-1	AL	L, RI	0.41
51	*α*-Farnesene	1738	502-61-4	T	RC	0.22
52	*δ*-Cadinene	1745	483-76-1	T	L, RI	0.32
53	2-Phenylethyl acetate	1798	103-45-7	E	RC	2.78
54	Methylethyl dodecanoate	1812	10233-13-3	E	L, RI	0.87
55	(*E*)-Geranylacetone	1819	37-96-70-1	T	RC	0.75

^1^ Retention index (polar DB-Wax fused silica capillary column 30 m × 0.25 mm i.d., 0.25 µm film thickness). ^2^ Chemical Abstract Service Number. ^3^ Group of a chemical compound. ^4^ Alkane. ^5^ Terpene. ^6^ Aldehyde. ^7^ Ester. ^8^ Alcohol. ^9^ Ketone. ^10^ Identification method. ^11^ Reference compound. ^12^ Mass spectra libraries (NIST and MassFinder3).

**Table 2:** Volatile compounds from headspace of sour cherry, *Prunus cerasus*, fruit and their electroantennographic activity to *Rhagoletis cerasi* fruit fly females.

No ^1^	Compound	RT ^2^	Amount		EAG ^4^ Activity	
			Mean ± SE ^3^	%	Mean ± SE ^5^	Number
1	(*E*)-2-Hexenal	8.3	12.95 ± 0.56	12.52	48.33 ± 14.03	7 ^6^ (12) **^7^**
2	Ethyl hexanoate	8.74	0.06 ± 0.01	0.06	11.67 ± 6.26	3 (12)
3	(*E*)-*β*-Ocimene	9.09	10.60 ± 0.28	10.25	36.37 ± 13.22	6 (12)
4	3-Methylbutyl 3-methylbutanoate	10.24	3.55 ± 0.08	3.43	36.67 ± 17.20	5 (12)
5	(*E*)-4,8-Dimethyl-1,3,7-nonatriene	10.46	24.11 ± 0.50	23.32	31.67 ± 12.42	10 (12)
6	Ethyl octanoate	13.66	35.09 ± 0.90	33.94	61.67 ± 16.60	7 (12)
7	1-Octen-3-ol	14.03	0.17 ± 0.04	0.16	48.33 ± 15.85	8 (12)
8	(*Z*)-3-Hexenyl 3-methylbutanoate	14.87	0.73 ± 0.01	0.71	65.00 ± 18.44	9 (12)
9	Linalool	16.36	3.47 ± 0.09	3.36	53.33 ± 8.99	9 (12)
10	Unknown	18.85	0.05 ± 0.01	0.05	11.67 ± 6.26	3 (12)
11	*α*-Muurolene	20.23	0.10 ± 0.01	0.10	30.00 ± 13.37	4 (12)
12	*α*-Farnesene	20.67	0.30 ± 0.01	0.29	28.33 ± 12.18	4 (12)
13	2-Phenylethyl acetate	21.88	4.81 ± 0.10	4.65	80.00 ± 21.46	8 (12)
14	(*E*)-Geranyl acetone	22.91	7.41 ± 0.30	7.17	76.67 ± 16.67	11 (12)

^1^ Number of a compound as indicated in Figure 1. ^2^ Retention time. ^3^ Value is an absolute amount expressed as area under a chromatographic peak and must be read as number times 10,000. ^4^ Electroantennographic. ^5^ Mean of EAG response of female antenna in µV. ^6^ Number of female antennae which responded to a stimulus. ^7^ Number of female antennae tested.

## Data Availability

The data presented in this study are available in article.
